# Invasive *Aspergillus* rhinosinusitis complicated with cerebral abscess

**DOI:** 10.1590/0037-8682-0296-2021

**Published:** 2021-08-20

**Authors:** Vee Vian Wang, Chee Yik Chang, Anuradha P. Radhakrishnan

**Affiliations:** 1 Department of General Medicine, Hospital Selayang, Malaysia.

**FIGURE 1:**
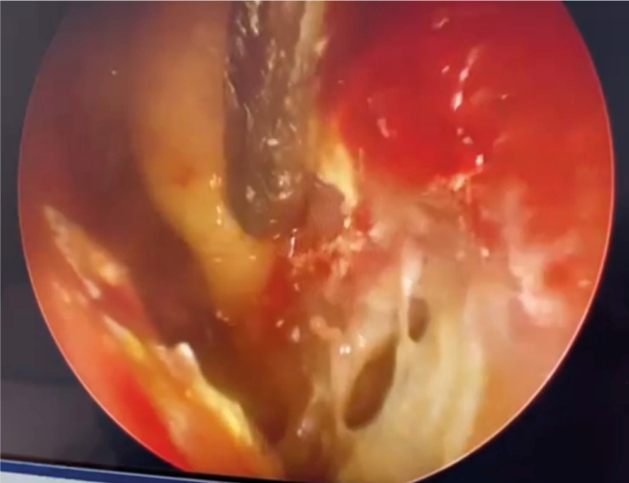
Nasal endoscopic image revealing right nasal septal wall perforations.

**FIGURE 2:**
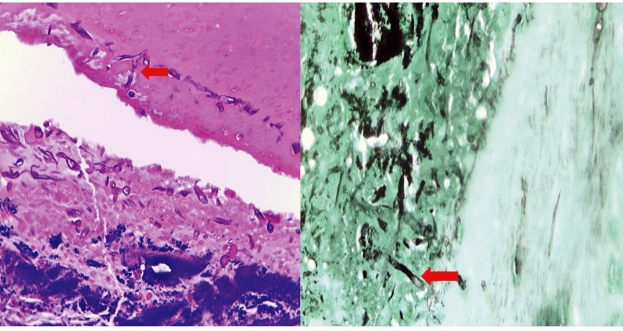
Histopathologic examination of the biopsied inferior turbinate showing broad septate fungal hyphae (arrow).

**FIGURE 3:**
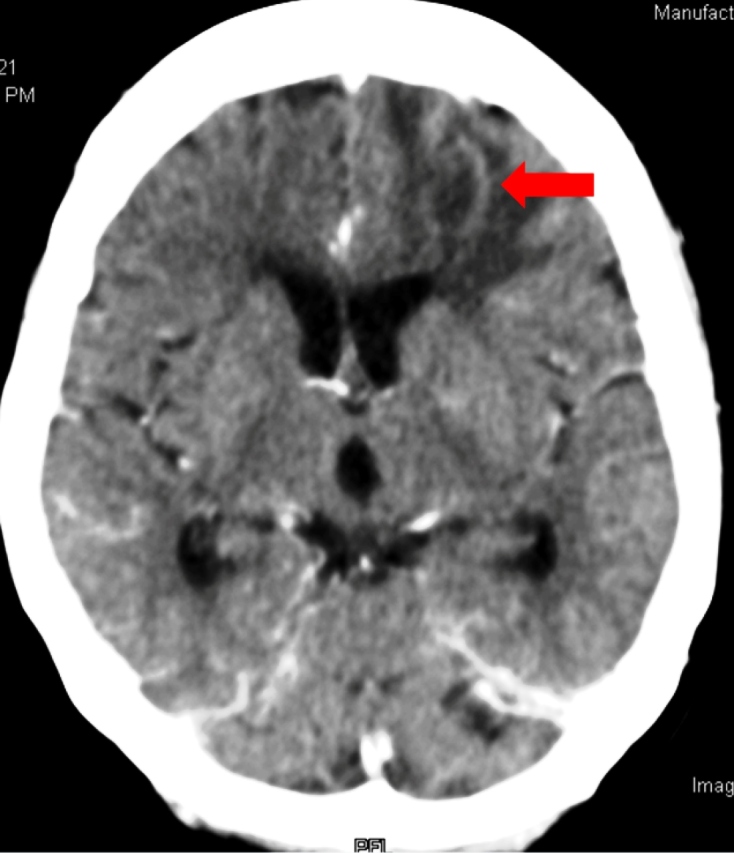
Computed tomographic scan of the brain showing abscess in the left frontal lobe (arrow).

A 66-year-old woman with poorly controlled insulin-dependent diabetes mellitus and rheumatoid arthritis and was on 10-mg oral methotrexate once-weekly had intermittent rhinorrhea-associated swelling in the right eye and cheek for four days. The symptoms started one week ago after thorough house cleaning. Computed tomography (CT) of the paranasal sinuses and orbits showed mucosal thickening of the bilateral frontal, ethmoid, sphenoid, and maxillary sinuses, suggesting sinusitis. Nasal endoscopy revealed right septal wall perforations and a fungal mass in the right maxillary cavity (Figure 1). Histopathological examination of the biopsied inferior turbinate revealed broad septate fungal hyphae (Figure 2); pan-fungal polymerase chain reaction showed *Aspergillus fumigatus*. Therefore, oral voriconazole was administered. She had decreased consciousness after three-week antifungal therapy. Urgent contrast-enhanced CT brain showed left frontal lobe abscess measuring 4.1×1.8×3.2 cm with a slight midline shift (Figure 3). However, her family refused neurosurgical intervention. Her condition deteriorated further and died one month later. 

*Aspergillus*, a mold, causes various diseases including pulmonary aspergillosis, rhinosinusitis, central nervous system (CNS) aspergillosis, endocarditis, osteomyelitis, and endophthalmitis. *Aspergillus fumigatus*, the primary causative agent of aspergillosis, accounts for 90% of human infections^1^. Risk factors for invasive aspergillosis include prolonged neutropenia, transplantation, prolonged and high-dose corticosteroid therapy, hematological malignancy, chemotherapy, and advanced acquired immunodeficiency syndrome^2^. CNS aspergillosis is rare *and* may manifest as single or multiple cerebral abscesses, meningitis, epidural abscess, or subarachnoid hemorrhage. Despite antifungal therapy, CNS aspergillosis has a poor prognosis, with mortality rates exceeding 90% owing to poor penetration of antifungal drugs into the CNS^3^.
